# Systemic and Local Silk-Based Drug Delivery Systems for Cancer Therapy

**DOI:** 10.3390/cancers13215389

**Published:** 2021-10-27

**Authors:** Anna Florczak, Tomasz Deptuch, Kamil Kucharczyk, Hanna Dams-Kozlowska

**Affiliations:** 1 Department of Cancer Immunology, Poznan University of Medical Sciences, 61-866 Poznan, Poland; annaflorczak@ump.edu.pl (A.F.); tdeptuch@ump.edu.pl (T.D.); kamilkucharczyk@ump.edu.pl (K.K.); 2 Department of Diagnostics and Cancer Immunology, Greater Poland Cancer Centre, 61-866 Poznan, Poland

**Keywords:** silk fibroin, spidroin, sericin, local drug delivery, systemic drug delivery, cancer therapy

## Abstract

**Simple Summary:**

Application of drug delivery systems (DDS) in oncology may increase the effectiveness of cancer treatment and reduce the associated adverse side effects. Although various biomaterials can be considered for the development of DDS, the materials of natural origin offer great biocompatibility and degradability. Silk is a natural biomaterial with exceptional properties, and one of them is the possibility to form diverse morphological structures. Scaffolds, films, hydrogels, fibers, foams spheres, capsules, microneedles, among others, can be used for local and systemic drug delivery. In this review, we described the various silk-based DDS for potential application in oncology. However, the unique silk properties combined with the possibility of their further modifications and blending open the gate to numerous potential biomedical applications, not only in the oncology field.

**Abstract:**

For years, surgery, radiotherapy, and chemotherapy have been the gold standards to treat cancer, although continuing research has sought a more effective approach. While advances can be seen in the development of anticancer drugs, the tools that can improve their delivery remain a challenge. As anticancer drugs can affect the entire body, the control of their distribution is desirable to prevent systemic toxicity. The application of a suitable drug delivery platform may resolve this problem. Among other materials, silks offer many advantageous properties, including biodegradability, biocompatibility, and the possibility of obtaining a variety of morphological structures. These characteristics allow the exploration of silk for biomedical applications and as a platform for drug delivery. We have reviewed silk structures that can be used for local and systemic drug delivery for use in cancer therapy. After a short description of the most studied silks, we discuss the advantages of using silk for drug delivery. The tables summarize the descriptions of silk structures for the local and systemic transport of anticancer drugs. The most popular techniques for silk particle preparation are presented. Further prospects for using silk as a drug carrier are considered. The application of various silk biomaterials can improve cancer treatment by the controllable delivery of chemotherapeutics, immunotherapeutics, photosensitizers, hormones, nucleotherapeutics, targeted therapeutics (e.g., kinase inhibitors), and inorganic nanoparticles, among others.

## 1. Introduction

Cancer is the leading global cause of mortality, and cancer incidence is rapidly increasing. Surgery and radiotherapy are the most effective and valuable treatments in eradicating localized and nonmetastatic tumors, but the disease spread throughout the body can be controlled only by chemotherapy. However, conventional chemotherapeutic agents are distributed randomly in the body, where they affect both cancerous and normal cells. This distribution limits the drug dose achievable within the tumor and results in suboptimal treatment due to excessive toxicities. In addition to off-target side effects, low water solubility, low bioavailability, and rapid clearance from circulation are common drawbacks of conventional small-molecule drugs. To overcome these shortcomings, many multifunctional targeted drug delivery systems (DDSs) have been proposed to enhance the efficacy of drug delivery and the final therapeutic outcome [1].

Local drug delivery offers dramatically higher drug concentrations in tumor tissues, while reducing harmful side effects to healthy organs and minimizing local tumor relapse [2]. Locoregional cancer treatment relies on the implementation of drug delivery vehicles for cancerous lesions. This strategy involves synthetic or natural polymer-based foams, wafers, fiber mats, and scaffolds. In this approach, mostly biodegradable matrices are proposed to avoid additional surgery for the removal of the biomaterial and to prevent a chronic immune response against foreign bodies [2]. Systemic drug delivery relies on nanomaterial-based DDSs, such as liposomes, micelles, dendrimers, and nanoparticles (NPs) that deliver therapeutic agents to cancer [3,4]. These DDSs offer enhanced pharmacokinetic parameters, such as high clearance value, large volume distribution, and greater bioavailability to cancer cells. Synthetic polymers are used predominantly for designing drug carriers. Polymers, such as poly(lactic-co-glycolic acid) (PLGA), poly(lactic acid) (PLA), poly(ethylene glycol) (PEG), poly(methyl methacrylate) (PMMA), poly(ethyleneimine) (PEI), poly(methylene malonate) (PMM), and polyesters have been used to form NPs [5]. The surface of polymeric NPs can be modified with various moieties, such as drugs and ligands, to provide multimodal treatment [5]. On the other hand, natural polymers, such as polysaccharides, lipids, proteins, and polypeptides, have also been employed [6]. The advantage of biopolymers in comparison to synthetic polymers is their ability to undergo enzymatic degradation in natural environments, accompanied by the release of nonhazardous byproducts that can also be eliminated biologically [6].

Among natural polymers, silks are considered to be excellent candidates for various biomedical applications, as they are biocompatible, biodegradable, nontoxic, and induce only a mild immune response [7,8]. The exceptional mechanical properties of silk, in addition to its compatibility with common sterilization techniques and simple preparation methods, make it a perfect biopolymer for a wide range of uses, including cancer therapy. Silk is a useful matrix for controlled drug delivery, as diverse silk-based formulations can be tailored for size, stability, drug loading, and release kinetics by simply changing the processes of material formation and/or post-treatment [9]. Moreover, active tumor targeting may be realized by the conjugation of silk structures with different targeting moieties, such as peptides, antibodies, and aptamers, that target particular epitopes expressed on the surface of cancer and cancer-associated cells [10,11].

Silks are fibrous proteins produced by a variety of invertebrates. Various silk structural formats are generated in nature, including the best-known fibers produced by most spiders and silkworms but also sheet-like and ribbon-like morphologies that are formed by the tarantula [12]. Moreover, several types of silk can be distinguished. This heterogeneous group of proteins not only differs depending on the origin (each animal species produces a different silk fiber) but also can produce several kinds of silk. Each type of silk fiber provides a different structural role in cocoon and web formation, nest building, egg coating, or lifeline formation, which is critical for the survival of the specimen [13,14]. The number of different silks that originated from spiders and silkworms is large, with only a few to date having obtained detailed sequences and organizations of protein domains. The best-characterized and most used are mulberry silk from the domesticated silkworm *Bombyx mori* [15], nonmulberry silk produced by *Antheraea mylitta* [16], and dragline silks derived from the spiders *Nephila clavipes* and *Araneus diadematus* [17,18]. Silks can be processed directly from nature or be produced biotechnologically in a heterologous expression system [12].

This review concisely outlines the various strategies for the use of silk as a DDS for cancer treatment. After a short description of the most studied silks and presentation of the advantages of using silk for controlled drug delivery, we focused on local and systemic silk-based DDSs dedicated to cancer therapy.

## 2. Silk Fibroin

Silkworm silk was obtained by the extraction of *B. mori* cocoons. Cocoons are composed of two types of proteins, namely, fibroin and sericin, that differ in structure and properties. Additional components, such as wax, pigments, sugars, mineral salts, and other impurities, are also present [19]. The simultaneous presence of fibroin and sericin decreases the biocompatibility of the silk biomaterial [20]. However, since sericins are water soluble, they and other impurities sticking to the silk can easily be removed in the degumming process. Degumming can be performed by boiling silk cocoons in water or by using acidic or alkaline buffers. In the absence of sericin, silk fibroin causes minimal inflammatory reactions [21]. Once degummed fibers are obtained, they are solubilized with the use of highly concentrated lithium bromide (LiBr) or other chaotropic salts [22]. The resulting regenerated fibroins can be subsequently processed to form various biomaterial morphologies, such as films, hydrogels, foams, scaffolds, particles, and coatings [23,24,25,26,27,28]. Although the regeneration process is relatively cheap and efficient, materials made of regenerated silkworm silks often require further modification and processing to gain suitable properties as biomaterials [27].

The silk fibroin (SF) obtained from the cocoon of *B. mori* consists of heavy (~325 kDa) and light chains (~26 kDa) held together by a disulfide bond [29,30]. The silkworm SF heavy chain has a modular structure containing large internal repetitive sequences flanked by shorter N- and C-terminal domains [31]. The SF light chain contains nonrepeating amino acid sequences and is relatively more hydrophilic and elastic, with little or no crystallinity [32,33]. This subunit adopts a globular conformation and provides the fiber with increased mobility [34]. The amphiphilic structure of the SF heavy chain is responsible for the remarkable mechanical properties of silk. The heavy chain consists of 12 hydrophobic, crystalline motifs that account for 94% of the sequence. They are principally composed of five alternating amino acids, glycine (46%), alanine (30%), serine (12%), tyrosine (5%), and valine (2%) [31]. They form a highly conserved GAGAGS motif and a less conserved GAGAGX motif (X = V or Y) [29,31]. These domains are separated by 11 hydrophilic, amorphous regions that contain negatively charged, polar, and aromatic residues that share a consensus sequence TGSSGFGPYVANGGYSGYEYAWSSESDFGT [29,31,35]. The glycine- and alanine-rich hydrophobic motifs form layers of antiparallel beta-sheet secondary structures. They are responsible for the self-assembly of SF. These strong physical interactions result in robust structures with a slow degradation rate and excellent mechanical properties. Conversely, the amorphous hydrophilic regions endow silk with elasticity.

In addition to extraction methods that result in regenerated SF, another approach to obtaining silk fibroin is its recombinant production. The genetic fusion of sequences derived from different proteins allows the generation of polymers with unique biophysical and biochemical properties. Silk elastin-like proteins (SELPs) are an example of bioengineered silkworm silk-based copolymers that contain multiple repeats of the GAGAGS motif from silk fibroin coupled with the GVGVP motif derived from the elastin sequence [36,37].

## 3. Silk Sericins

Silk sericins (SSs) are mostly discarded in the processing of raw silk cocoon wastewater [38]. However, collected and recovered sericins can also be used as biomaterials. SS has been reported to be minimally inflammatory in the absence of fibroin [39,40,41], which suggests that the interaction of SS with SF may be related to inflammatory outcomes [42]. Despite this fact, sericins have been increasingly utilized in biomedicine due to their valuable properties, including enhanced biodegradability, biocompatibility, and cell adhesion. However, the potential of SS for the development of nanomedicines has not been investigated in detail. The physicochemical instability (at various pH values and temperatures) and high water solubility of SS limit its potential application [22]. On the other hand, to overcome the mentioned difficulties, SS can be combined with other polymers to develop DDSs. For this reason, SS has been blended with nonionic surfactants F-127 and F-87 [43], chitosan [44], poly(c-benzyl-L-glutamate) (PBLG) [45], hydroxyapatite (HAp) [46], or cholesterol [47].

Silk sericins are coating proteins that envelop the fibroin fiber. The sticky layers of SS help in the formation of a cocoon. Sericins constitute approximately 15–35% of the total cocoon weight [19,22]. Three types of SS proteins with different solubilities and amino acid sequences can be distinguished. Sericin A constitutes the external layer of the cocoon that can be easily removed by degumming silk cocoons in hot water. The middle space is occupied by sericin B, which has lower polarity than sericin A, despite the same amino acid composition. Finally, in the inner layer of the cocoon, in proximity to the SF filaments, sericin C is located, which indicates poor water solubility [48]. To remove all these sericin layers thoroughly, alkaline solutions must be used during the degumming process [48].

Sericins are globular proteins (20 kDa to 310 kDa) containing abundant polar side chains made of hydroxyl, carboxyl, and amino groups that provide SS with high chemical reactivity [38,40]. A total of 17–18 types of amino acids form the SS, but particularly high contents of hydrophilic amino acids, such as serine (37%) and aspartic acid (16%), are observed [40]. The SS secondary structure retains a combination of β-sheets and random coil domains, although the latter is often predominant. Crosslinking with glutaraldehyde or exposure to organic solvents (e.g., ethanol) induces sericin crystallinity, increasing the mechanical properties of SS [49].

## 4. Spider Silk

In contrast to silkworms, spiders cannot be farmed due to their cannibalistic and territorial nature. Moreover, collecting silk from webs is a time-consuming and relatively inefficient task. Spiders produce different types of silk simultaneously; thus, harvesting one kind of silk is a complex issue. Each spider silk protein (spidroin) differs in its primary sequence, has distinct properties, and is used for different purposes [14].

Among spider silk fibers, dragline silk is the most extensively studied, and the best characterized. The dragline silk is used as safety lines or as material to build web frames (anchors) [50]. Recently it was proposed that a safety line consists of two types of silk fibers: one (2–5 μm in diameter) formed in the major ampullate gland and the second one (1–3 μm in diameter) produced in minor ampullate gland [51]. Although both types of silk fibers are present in the safety line, they are used to build different parts of the web.

The *N. clavipes* dragline silk, produced in the major ampullate gland, consists mainly of two proteins: major ampullate spidroins 1 and 2 (MaSp1 and MaSp2, respectively) [52,53]. These spidroins generate complexes with a molecular weight of approximately 350 kDa [54]. Another spider, *A. diadematus*, produces dragline fiber composed of the *A. diadematus* fibroins 3 and 4 (ADF-4 and ADF-3, respectively) [55]. Similar to silkworm silk, most spider silk proteins are block copolymers composed of an extended repetitive region flanked by nonrepetitive regions at the N- and C-termini [54,56,57]. The N- and C-terminal domains (of approximately 130 and 110 amino acids, respectively) are involved in assembling and processing silk fibers. They provide charge-dense regions to facilitate aqueous solubility and modulate the self-assembly of silk induced by pH changes [58,59]. The core of the silk sequence, composed of repetitive sections, is responsible for its mechanical properties [51].

Analysis of the amino acid composition of dragline silks revealed that it consists mainly of glycine and alanine [60]. Within the repetitive core, amino acids can be grouped into four structural motifs: (i) polyalanine (poly-A), (ii) glycine–alanine (poly-GA), and glycine-rich domains (iii) GGX, and (iv) GPGXX [60,61,62]. The alanine-rich chains that form antiparallel beta-sheet nanocrystalline domains are responsible for the mechanical strength of the polymer [63,64]. Glycine-rich motifs that separate the poly-A and poly-GA regions form the noncrystalline or amorphous segments. The GGX motif forms the 3_1_-helical structures, and the GPGGX region forms type II β-turns, the repetition of which result in the formation of an extensible β-spiral [65,66,67]. The helical and turn structures are responsible for the elasticity of the silk material.

The MaSp1 does not contain the GPGGX motif in contrast to the MaSp2. In MaSp2 spidroin, the proline residues account for 15% of the total amino acid content [50]. Based on the number of proline amino acids in dragline silk, it is possible to assess the fiber content, which differs depending on the spider species. The major ampullate spidroins ratio affects the silk fiber properties. In *N. clavipes* dragline silk, the MaSp1:MaSp2 ratio is 81:19% [68]. MaSp1 is found uniformly in the fiber’s core, whereas MaSp2 is in homogeneously distributed along with silk fiber; it is missing in the periphery of the fiber core and forms clusters in certain core areas [69].

The ADF-4 and ADF-3 spidroins both contain proline residues; thus, their presence is not a good indicator of the content of a given protein type. These proteins differ in intrinsic characteristics, such as hydropathicity; ADF4 being more hydrophobic while ADF3 being more hydrophilic [70]. The same can be observed for major ampullate silks of different species, which constitutes a major difference between these two types of proteins. In *N. clavipes* dragline silk, MaSp1 displays relatively high hydrophobicity, whereas the MaSp2 proteins are more hydrophilic [70]. The characterization of bioengineered silk proteins based on these two dragline silk-type proteins revealed that physicochemical properties, such as charge and hydrophobicity greatly impact their self-assembly performance [70,71].

For further details on the structures and mechanical properties of various silk types from different spider species, the reader is directed to excellent recent reviews [61,72,73].

As mentioned above, harvesting naturally occurring spider silk of repeatable quality is a nearly impossible task. However, the recombinant production of bioengineered spider silks allows us to resolve the problem of the quality and quantity of spider silk. The recombinant DNA approach enables the achievement of the desired sequence of silk, which determines its structure and properties. The process of recombinant silk production consists of several stages, including (i) design and construction of a synthetic silk gene, (ii) ligation of the gene insert into an expression vector, (iii) transformation of the host cells with a vector carrying the silk gene, and (iv) protein expression and purification. Based on the amino acid sequence of natural spider silk, short oligonucleotide sequences corresponding to silk monomers can be designed and synthesized. The application of gene multimerization techniques enables the construction of large repetitive sequences composed of multiple silk monomer units. Concatemerization, the ligation of DNA monomers having complementary cohesive ends, allows the generation of a library of genes of various sizes in a single-step process. However, the precise control of the preparation of genes with a specific composition and size using this method is limited. Recursive directional ligation or step-by-step ligation are cloning strategies that can overcome the limitations of concatemerization [74]. The application of these methods enables precise control of the size of the obtained gene. In recursive directional ligation, DNA monomers of one kind are directly self-ligated or ligated with monomers of other sequences, which eliminates restriction enzyme sites between them. This method results in a silk gene without any external insertions in the sequence. Step-by-step ligation also involves the self-ligation of one or various silk DNA monomers; however, the resulting gene sequence contains fragments encoding restriction enzyme sites between silk monomers. Various recombinant spider silks and silk-like proteins, which were based on *Nephila clavipes* dragline silk sequences (MaSp1 and MASp2), were obtained using a step-by-step directional approach [73,75]. Huemmerich et al. demonstrated the construction and production of recombinant silks derived from *Araneus diadematus* dragline proteins, such as ADF3 and ADF4 [76]. The seamless and controlled assembly of multiple silk coding gene modules was shown. The single monomers were ligated, gradually multimerized, and optionally linked with nonrepetitive regions [76]. The obtained synthetic silk gene constructs can be subsequently ligated into expression vectors. A variety of heterologous host systems have been explored to produce recombinant silks, including bacteria, yeast, insects, and mammalian cells, as well as transgenic plants and animals (reviewed in [75,77,78]). Due to its ease of manipulation, short generation time, low cost, and ability to scale up the production process, *E. coli* bacteria are the most widely used host for the expression of silk [75].

The primary advantage of recombinant spider silk production is the homogeneity of the obtained polymer. Furthermore, since genetic engineering allows the design of synthetic genes, it is possible to further extend the already excellent silk properties for more customized applications. The genetic functionalization of silk can result in modification of its amino acid composition or the addition of a peptide or protein that determines a function. For drug delivery applications, the carriers should controllably bind/release a drug and selectively recognize the targeted cells. By adding polylysine or polyarginine blocks to spider silk sequences, improved cellular uptake of the obtained silk spheres was observed [79,80,81]. These modifications also allowed the binding of therapeutic nucleic acids [80,81]. Enhanced internalization into cells of spider silk particles can also be achieved by the functionalization of bioengineered silks with integrin binding motifs (RGDs) or different cell-penetrating peptides (CPPs), such as transactivator of transcription (Tat) or ppTG1 peptide [82,83,84,85,86]. Another approach that enables increased selectivity involves the combination of silk proteins with tumor-homing peptides (THPs) that recognize particular molecules on the cancer cell surface [87]. Among others, the successful fusion and formation of various structures made of silk was described for (i) F3 peptide, which binds specifically to nucleoin expressed on the surface of angiogenic endothelial and some tumor cells, (ii) CGKRK peptide, which binds to heparan sulfate in tumor vessels, and (iii) Lyp1 peptide, which targets the lymphatic vessels of certain tumors [84,85]. Due to its overexpression in invasive breast carcinomas and other neoplastic transformations, human epidermal growth factor receptor 2 (Her2) has become a target for anticancer therapy. Spheres made of bioengineered spider silk functionalized with Her2 binding peptide (H2.1MS1) were efficiently internalized and transported doxorubicin into Her2-positive cancer cells [88,89]. The specific binding and accumulation of H2.1MS1 spheres in Her2-overexpressing tumors were observed in vitro and in vivo [89,90]. The intravenous administration of doxorubicin-loaded particles caused the inhibition of tumors in both primary and metastatic breast cancer models [90]. The silk functionalization strategies have been summarized in detail recently in other review articles [10,91].

## 5. Advantages of Silk Proteins for Controlled Drug Delivery

**Figure 1:**
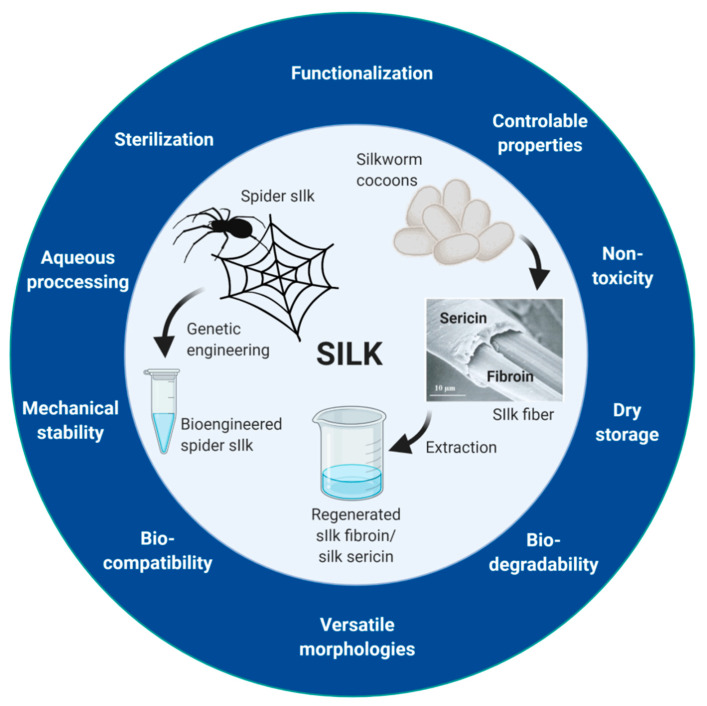
Key advantageous properties of silk proteins for biomedical applications and overview of the origin of silk proteins. Picture presenting the composition of silkworm silk fiber was reproduced with permission [103]. Created with BioRender.com accessed on 28 June 2021.

Silks have many characteristics that make them a promising material for biomedical applications (Figure 1). Silks are biocompatible and enzymatically biodegradable [92,93,94,95,96,97]. Furthermore, silk materials are nontoxic and have low immunogenicity [7,98,99,100,101]. Finally, silk materials have excellent mechanical stability and a controllable format and size, and they can be stored in a dried state because of their reversible swelling behavior [102], offering unlimited opportunities for the fabrication, functionalization, and processing of robust biomaterials.

As mentioned above, silks’ physicochemical properties are the consequence of the primary and secondary structure of silk proteins. The highly repetitive hydrophobic crystalline regions interspaced with the hydrophilic noncrystalline blocks provide a unique hierarchical structure. They are responsible for silk protein self-assembly, leading to strong physical interactions and robust mechanical structures that result in biomaterial strength and toughness [9]. Moreover, the presence of hydrophobic domains in silk (both the GAGAGS motif in silkworm silk, and poly(GA) and poly(A) sequences in spider silk) determines the crystalline content. These domains enhance the hydrophobic carrier-drug interactions, allowing control of the loading and release of drugs [9,104]. The use of genetic engineering to build synthetic silk genes allows the regulation of the content of crystalline and noncrystalline blocks, which can translate into additional control of the mechanical properties of silk and its interaction with the drug.

A key issue with adjusting the biomaterial performance is control over the processing conditions of silk proteins (self-assembly) in aqueous solution without chemical additives [105,106]. It is possible to modulate the degree of exposure of silk crystallinity by methanol or water vapor annealing [107]. Treatment with methanol or water vapor induces β-sheet formation and controls diffusive pathways, thus reducing the initial drug burst release and extending the release of drugs [107].

The crystalline content influences not only the mechanical properties of silk and drug entrapment but also the degradation profile of the silk formulations; the lower β-sheet crystal content and higher helical content in the silk biomaterial, the degradation rate is significantly faster compared to the crystal-rich silk material [108]. Importantly, control over the degradation profile of silk polymers enables adjustment of the degradation-based drug release from silk materials and achieves sustained drug release [104].

The material property may also be controlled by blending the other polymers or substances with silk [43]. This approach to DDS design enables combining materials of different origins to exploit benefits from both of them. For example, SF/albumin nanoparticles were developed for the delivery of chemotherapeutics. The presence of albumin in the nanosystem improved the mechanical properties and biodegradability of SF-based particles [109]. Moreover, combining silk with iron oxide nanoparticles resulted in pH-dependent, advantageous kinetics of chemotherapeutic release (limited drug release in the blood and enhanced drug release into a tumor site) [110].

On the other hand, the solubility, stability, molecular weight, and charge of drug substances must be considered in designing suitable carrier systems [111]. It has been shown that model drugs or low molecular weight molecules can be loaded into silk particles in various amounts, depending on the physicochemical properties of the loaded substance [112,113,114]. The predominant hydrophobic nature of silk determines the characteristics of therapeutic compounds that are incorporated into the silk carrier; therefore, hydrophobic drugs usually perform better in terms of drug-silk interactions and sustained release behavior [115]. However, drug incorporation may also be achieved by electrostatic interactions between negatively charged silk nanoparticles and positively charged drugs (or vice versa). Negatively charged molecules are generally released faster than positively charged molecules, apparently due to electrostatic repulsion [114,116]. To control the electrostatic interaction with drugs, the genetic functionalization of silk can be applied. By adding charged amino acids or peptides (such as lysine, arginine, aspartic acid, and glutamic acid), the zeta potential of silk-based DDSs was modified [80,82,113,117,118].

Another essential feature of silk as a biomaterial for controlled delivery is the presence of reactive amino acids (AAs) containing multiple side groups. Such AAs can be used for the addition of unique chemical moieties [119]. Control over the degree of silk functionalization is facilitated by exploiting the chemical groups of amino acids, such as tyrosine and glutamic acid [119]. Although SF lacks cell-instructive cues (e.g., sequences for cell adhesion and peptide sequences for targeting), the presence of the reactive AA allows chemical modification to tailor the protein for the desired application. These modifications provide chemical handles for the attachment of cell-binding domains, growth factors, and other polymers to silk [21,119]. The SF nanoparticles were covalently decorated with the integrin-recognition sequence Arg-Gly-Asp (RGD motif) to increase their ability to target intestinal tissue [120]. In another approach, silk proteins were chemically modified with a hydrazone linker, which led to a pH-responsive carrier system. Such a modification allows enhanced drug release from silk carriers in the acidic microenvironment, e.g., in tumorous tissues [121].

Moreover, the possibility of modifying bioengineered silks allows the design of drug delivery vehicles with tunable features. For example, the fusion of sequences derived from silkworm silk fibroin (multiple repeats of the GAGAGS motif) and the elastin sequence (the GVGVP motif) generated silk elastin-like proteins (SELPs) [36,37]. In this fusion construct, the silk component is responsible for the biomaterial’s strength and ability to self-assemble into higher structures, while the elastin component provides control over the material’s physical state (liquid vs. solid) [37]. The combination allows us to obtain an injectable drug delivery system [122,123].

Moreover, as mentioned above, genetic engineering enables the incorporation into silk of a sequence that determines a function that can provide an opportunity to adjust silk materials for more customized applications. Bioengineered functionalized silk-based systems were explored, that provided control over drug delivery to selected cells (targeted delivery) [82,83,84,85,88,89,90,124], chemotherapeutic binding/release [117,125], binding of inorganic nanoparticles [126], binding of therapeutic nucleic acids [80,82,83,84,85], and binding of photosensitizing agents [127].

The final advantageous feature of silk is the versatility of options for sterilization. The filter sterilization of silk solution can be applied, and then aseptic silk can be used for particle formation [128]. Moreover, several common sterilization methods can be implemented in terms of a silk biomaterial, due to their high mechanical and thermal stability [129]. Silk-based materials can be sterilized by standard autoclaving, gamma radiation, or ethylene oxide. Hedhammar et al. demonstrated that fibers made of spider silk could be steam autoclaved, while preserving their morphology, structure, and mechanical properties [130]. The steam sterilization did not affect the size, secondary structure, or thermal stability of particles made of the bioengineered spider silk eADF4(C16) [131]. Hofmann et al. studied the influence of various methods of sterilization, i.e., autoclaving (121 °C, high-pressure steam), dry heat (180 °C), ethylene oxide (55 °C), or exposure to disinfecting agents (70% aqueous ethanol or an antibiotic-antimycotic solution) on silk fibroin scaffolds properties [132]. The results showed that the sterilization affected to a minimal extent the morphology, topography crystallinity, and cytocompatibility of analyzed scaffolds. On the other side, Rnjak-Kovacina et al. demonstrated that depending on the sterilization technique, the properties of silk biomaterials may be different [128]. The application of autoclaving caused the increase in silk fibroin scaffold stiffness and decreased degradation rate, while gamma irradiation accelerated its degradation. The adhesion and proliferation of human fibroblast cells on fibroin scaffolds were reduced after the ethylene oxide treatment and improved when the material was autoclaved [128]. Moreover, an increased gamma irradiation dosage caused an increase in the degradation of silk-based biomaterials [133]. The application of irradiation may also influence the mechanical properties of silk fibroin materials. It was shown that the UV-irradiation and γ-irradiation caused a decrease in tensile properties of respective spider silk fibers [134]. After γ-irradiation treatment, the decline in thermal stability and decreased tensile strength of degummed *B.mori* silk fibers were also observed [135]. Although various sterilization techniques can be applied, the final choice of the sterilization technique should depend on the intended application and desired silk-based material properties.

## 6. Silk-Based Biomaterials for Drug Delivery in Cancer Treatment

**Figure 2:**
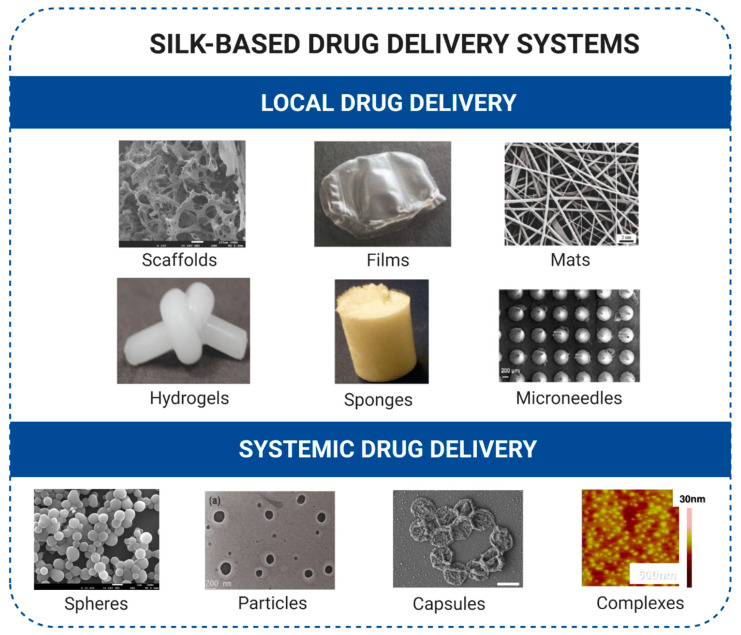
Silk-based systems used for local and systemic controlled delivery of therapeutic agents. Images are reproduced with permission from the cited articles for scaffolds [141], film [142], fiber mats [143], hydrogels [144], sponges [128], microneedles [145], spheres [125], particles [146], capsules [147], and complexes [83]. Created with BioRender.com accessed on 31 August 2021.

The vast majority of anticancer agents are poorly soluble in water; hence, a biomaterial carrier that allows the binding and release of these drugs would improve drug bioavailability and contribute to better therapy outcomes. The controllable processability of silk material into different morphologies, such as films, hydrogels, particles, sponges, scaffolds, or nonwoven meshes, can be advantageous for exploring the different administration routes of drugs. In anticancer therapies, silk-based drug delivery systems can be used locally by intratumoral and transdermal administration or systemically by intravenous injection [23,90,136,137,138,139,140]. The delivery of different classes of therapeutic molecules has been explored using a variety of formats of silk biomaterials. Figure 2 summarizes the various morphological structures made of silk for the local and systemic delivery of anticancer drugs.

### 6.1. Local Drug Delivery

For local drug delivery purposes, various two- and three-dimensional silk-based material formats have demonstrated great control over drug release rates [24,25,26,140,148,149].

Silk structures in two dimensions (2D) include films, coatings, and fiber mats. Silk films are optically transparent matrices typically generated by casting an aqueous or organic solvent-based SF solution and air-drying [150]. However, other preparation techniques can be applied, such as spin coating [151], vertical deposition [152], or layer-by-layer assembly [153]. Solvent treatments (e.g., immersion in alcohol or kosmotropic salts) or water annealing enable tuning of the ratio of the silk crystallinity [151,154]. As mentioned above, the content of specific secondary structures in SF-based films or coatings affects the release rate of the drug. By controlling the thickness of film/coating layers as well as the number of layers, it is possible to regulate the drug release rate from the material [155]. In the context of anticancer therapy, Seib et al. implemented silk films loaded with doxorubicin in a model of breast cancer [25]. The locally administered films demonstrated sustained drug release over 4 weeks that could be controlled by manipulating the silk crystallinity and beta-sheet content. Doxorubicin-loaded silk films significantly inhibited primary and metastatic tumors without any associated toxicity compared to intravenously administered free doxorubicin [25]. Two-dimensional nanofibrous mats are typically fabricated using the electrospinning technique [156]. With this technology, additional post-treatment of electrospun silk mats can improve the mechanical properties of the material by increasing the β-sheet content, crystallinity, and number of aligned fibers [157]. For instance, curcumin-loaded silk nanofibrous matrices were examined in terms of the long-term response of the human colorectal cancer cell line HCT-116. The local implantation of drug-loaded formulations enhanced the anticancer effect and resulted in tumor growth inhibition in vivo [158].

Three-dimensional (3D) silk implants include injectable hydrogels, foams, and porous scaffolds and sponges. Hydrogels are chemically or physically cross-linked, involving water-containing 3D networks that swell but do not dissolve when brought into contact with water. Silk-based hydrogels can be produced by the sol-gel transition in the presence of acid, ions, or other additives [26,159]. Porous sponges and scaffolds made of silk fibroin are produced from the aqueous solution of SF by inducing a gelation reaction. The pore architectures and sizes of porous scaffolds/sponges can be manipulated by controlling the protein concentration and freezing temperature, utilizing gas foaming, dehydrating agents, or porogens [21]. Moreover, 3D printing employing extrusion dispensing devices can be exploited to fabricate 3D silk-based scaffolds or sponges [160]. However, silk scaffolds are robust, but they lack deformation, thus limiting their potential applications. Unlike salt-leached sponges, silk-based foams prepared by a foaming method are susceptible to deformation and more suited for smaller, injectable filling needs [161,162]. Local delivery of chemotherapeutics using 3D silk-based formulations has been evaluated in numerous studies [23,24,26,138,149]. Seib et al. prepared self-assembling silk fibroin hydrogels that showed no swelling and were readily loaded with doxorubicin under aqueous conditions [26]. The gelation process was found to be affected by many parameters, such as silk fibroin concentration, temperature, and pH value. The drug-loaded hydrogels exhibited sustained drug release over 4 weeks in amounts that could be tuned by varying the silk content. This approach enabled the inhibition of primary and metastatic tumor growth and reduced drug-associated toxicity [26]. Coburn et al. described a new intratumoral treatment strategy using implantable doxorubicin-loaded foams. The silk-based foams indicated sustained drug release for up to 25 days, and allowed significant tumor growth inhibition in a mouse model of neuroblastoma [23].

Transdermal administration is a local route of drug delivery that avoids degradation of the drug in the gastrointestinal tract [163]. For the transdermal administration of the drug, SF-based structures such as microneedles have been used [145,164]. Microneedles are produced by using numerous methods, including micromolding, soft lithography [164], photolithography, droplet-born air blowing, solvent casting, continuous liquid interface production, and dipping [137]. Microneedles made of biocompatible polymers enable drug loading in the needle matrix and spontaneous drug release after skin penetration via polymer swelling and dissolution [137]. Silk microneedles are a relatively simple, minimally invasive, and painless approach to delivering drugs across the skin. Moreover, similar to other silk-based material formats, they offer mild aqueous processing conditions, biocompatibility, controllable biodegradation, and robust mechanical properties that are sufficient to penetrate the skin for the delivery of pharmaceuticals followed by degradation and excretion from physiological environments [15,155]. The most frequently explored approaches using silk-based composite microneedles are transdermal vaccines [165,166] and contraceptive delivery [145]. However, in a study by Gao et al., silk fibroin microneedles were capable of transporting the loaded doxorubicin and photothermal compound across the skin without causing pain [137], which holds future promise for the delivery of a broad range of biomolecules in cancer therapies.

Table 1 summarizes various silk fibroin formulations for the local delivery of therapeutic agents in cancer treatment.

### 6.2. Systemic Drug Delivery

Injectable silk-based formulations, such as capsules, spheres, and particles, have been used for the systemic administration of various therapeutic molecules. Silk-based capsules are fabricated via layer-by-layer deposition of ingredients over a template of size range varying from nm (nanocapsules) to μm (microcapsules). In this approach, the template dissolves, and hollow capsules are produced that enable the entrapment of drug molecules inside the capsules [172]. Silk micro- and nanospheres have been developed as an active depot drug delivery system [173]. The diffusion of drug molecules through the polymer network and/or material degradation determines the drug release from such spheres [173]. Silk particles are spherical, stable, and mostly negatively charged vehicles [155]. They typically possess a high surface-to-volume ratio, high carrying capacity for the entrapment of bioactive molecules, and the ability to deliver them to target sites [21]. A wide range of manufacturing strategies have been used to generate silk particles, which are discussed below. Silk injectable nano and microformulations can be obtained based on regenerated silk fibroin, sericin, and bioengineered silk proteins [9,22,77]. Independent of silk origin, these vehicles are excellent carriers for the delivery of bioactive molecules due to their superior mechanical properties, such as high elastic modulus and toughness [155]. Moreover, nanosized DDSs are able to penetrate through small capillaries across physiological barriers and be incorporated into cells. Therefore, silk-based nanoparticles, nanospheres, or nanocapsules for drug delivery have been extensively studied for treating various diseases, including cancer [90,136]. On the other hand, microsized particulate systems are also used as depot drug carriers for long-acting delivery, and they are usually administered intramuscularly or subcutaneously [104].

The systemic drug delivery studies for cancer treatment, demonstrating the delivery of chemotherapeutics and other anticancer therapeutic agents using silk-based nanoparticles are presented in Table 2 and Table 3, respectively. We summarized the physical properties of silk particles and the most important findings related to the use of in vitro and in vivo drug delivery systems in cancer models (Table 2 and Table 3).

## 7. Preparation of Nanoparticles Made of Different Silk Proteins

In nature, silks are produced in an elaborate and complex process in the highly specialized glands of silk-producing organisms. The unique physiological conditions provided by the silk glands allow individual silk proteins to undergo rapid self-assembly into fibers [213]. Although the natural process remains quite challenging to replicate in laboratory settings, some methods of silk material formation have been proposed that also rely on the ability of silk protein to self-assemble into higher structures [214]. Furthermore, those methods allow obtaining materials that display morphologies other than fibrous morphology, e.g., scaffolds, films, hydrogels, micro- or nanoparticles. For systemic drug delivery, nanoparticles are of particular importance. There are a variety of manufacturing strategies for the formation of silk nanoparticles, such as self-aggregation [109], desolvation [146,174,175], salting out [116,178], microfluidics [215], electrospraying [176,188,216], microemulsion [217], ionic liquids [218], sol-gel techniques [219], laminar jet break-up [107], supercritical fluids [220], and milling technologies [221,222]. In general, the formation of stable silk nanoparticles requires a change in the silk secondary structure conformation from random coils to physically crosslinked β-sheets due to an increase in protein molecule packing.

In the desolvation method, the self-assembly of silk particles is initiated by the liquid-liquid phase separation of the silk from the solvent (aqueous phase). The addition of a desolvating agent reduces the solubility of the silk, which promotes its aggregation and the formation of β-sheet structures between adjacent silk molecules. Due to the high content of β-sheet structures, silk aggregates fall out of solution, producing spherical structures. Commonly used desolvating agents include protonic organic solvents (e.g., methanol, ethanol, propanol, or isopropanol) or aprotonic organic solvents (e.g., acetone or DMSO) [223]. The main advantages of this method are simplicity and the relatively mild silk-processing conditions.

Salting-out effects can also be exploited to induce the self-assembly of silk molecules into particles. The salt concentration affects the solubility and stability of proteins in the solution. While a low concentration of salt often stabilizes proteins in the solution, a significant increase in its concentration might induce the salting-out effect, as the solubility of silk proteins is lower at high salt concentrations due to electrolyte–nonelectrolyte interactions [224]. High concentrations of salt ions disrupt the hydration layer of the silk, promoting protein–protein interactions and consequently their aggregation and precipitation. Kosmotropic salts display strong bonding interactions with water molecules, promoting salting-out effects, and have often been used for the preparation of silk particles [224]. Mixing silk proteins with kosmotropic agents induces the formation of silk protein coacervates, the nucleation process of the particles, and the transition of amorphous silk structures into β-sheet structures, which finally results in the generation of stable silk spheres [225]. For the formation of particles based on bioengineered spider silk, the most commonly used approach is the salting-out of silk with a highly concentrated potassium phosphate buffer [106]. The silk spheres can be formed either via the slow process of dialysis or rapid mixing. In the first method, the silk solution is dialyzed against potassium phosphate buffer [106], whereas the mixing process can be executed with a pipette or within a micromixing device with laminar or turbulent flow [106,226]. Several parameters determine the particle size, including the concentration of the protein solution and phosphate buffer, as well as mixing speed and mixing time [106,226]. A small and uniform particle size is of great importance for drug delivery applications. In general, the silk sphere diameter decreases with decreasing protein concentration, increasing phosphate buffer concentration, and increasing mixing speed [106,226]. Manual-based nanoparticle preparation is an easy and robust method. However, microfluidic manufacturing routes are now being explored to optimize, scale up, and control the silk nanoparticle production process and enhance product quality and repeatability [226,227].

Silk fibroin particles were also achieved using a microemulsion method [217]. Microemulsions are stable nanosized droplets formed as a result of mixing two immiscible fluids in the presence of surfactants (e.g., water-in-oil microemulsion) [228]. The addition of water-soluble silk proteins to cyclohexane in the presence of surfactant (Triton X-100) resulted in the formation of microemulsion templates entrapping soluble silk proteins [217]. The microemulsions were then recovered with methanol/ethanol and finally dialyzed against water. The methanol/ethanol treatment induced self-assembly and the formation of crystalline β-sheet structures in the silk particles entrapped in the microemulsion templates through dehydration of the silk proteins. Furthermore, the presented method also allowed for efficient entrapment of fluorescent dye (rhodamine B) inside the silk particles [217].

Some of the recent protocols of particle micronization utilize supercritical fluid technology. Supercritical fluids are substances whose temperature and pressure are above their critical point at which the gaseous and liquid phases are indistinguishable. Supercritical CO_2_ (scCO_2_) is one of the most commonly used supercritical fluids because it easily achieves its critical conditions (Tc = 31.1 °C, Pc = 7.38 MPa) and is relatively inexpensive, nonflammable, and nontoxic. In the preparation of silk particles, two main methods utilizing scCO_2_ have been adopted: the supercritical antisolvent (SAS) technique and solution-enhanced dispersion by supercritical fluids (SEDS) [197,220,229,230]. In the SAS method, the silk fibroin is dissolved in solvent and atomized in the scCO_2_ environment in a high-pressure vessel. The mass transfer of scCO_2_ (antisolvent) to the droplets removes the solvent, induces supersaturation of the sprayed substance, and causes the solute to fall out of the solution, generating nano- and microparticles. This method has been used by Chen et al. to generate indocyanine green-encapsulated silk fibroin (ICG-SF) nanoparticles that may find an application in phototherapy [229]. The SEDS method is a minor modification of the SAS process. Introduction of the special coaxial nozzle ensures improved mixing between organic solution (e.g., soluble silk) and scCO_2_, allowing better control over the size and morphology of generated particles. The SEDS method was used for the preparation of silk nanoparticles that could entrap active substances (e.g., curcumin) [197,220,230].

Electrospraying can also be used to generate silk particles [188,216]. This method provides liquid atomization of the solution by electrostatic forces. As the silk solution flows through the capillary nozzle, a high electric potential is applied, resulting in the formation of uniform droplets that are evenly dispersed on the grounded collector plate [216] or into the collection bath containing liquid nitrogen [188]. In the first method, sphere formation is induced by solvent evaporation, which triggers silk protein aggregation and the formation of spherical structures [216]. In the second method, spheres are obtained by freeze-drying electrosprayed silk solution [188].

Silk particles can also be obtained through the mechanical processing of *B. mori* cocoons [206]. Subsequent grinding or milling steps reduce the size of the generated particles. The major limitation of this preparation method is the wide size distribution of the generated particles [206].

Most of the methods for the preparation of silk spheres can demonstrably incorporate both hydrophobic and hydrophilic drugs into carriers [43]. Generally, different strategies can be used to load therapeutic molecules into silk-based nanoparticles. These drug loading approaches include the preloading method, also called entrapment or encapsulation, which is achieved by mixing the drug solution with silk protein prior to carrier formation [114]. The absorption method is a diffusion-driven procedure, also known as the coincubation technique or postloading method. This method is based on the incubation of drugs with obtained particles. This diffusion-driven method is based on the electrostatic interaction between the negatively charged silk nanoparticles and the positively charged drug molecules [114,116,174]. The second factor determining diffusion-driven drug loading is associated with strong hydrophobic interactions of silk biopolymer with drug molecules. Finally, different crosslinking agents, such as glutaraldehyde, ethylcarbodiimide and polyethylenimine, or physical factors (e.g., UV) can also be implemented for silk–drug interactions and the formation of covalent bonds [44,189,191,231].

**Figure 3:**
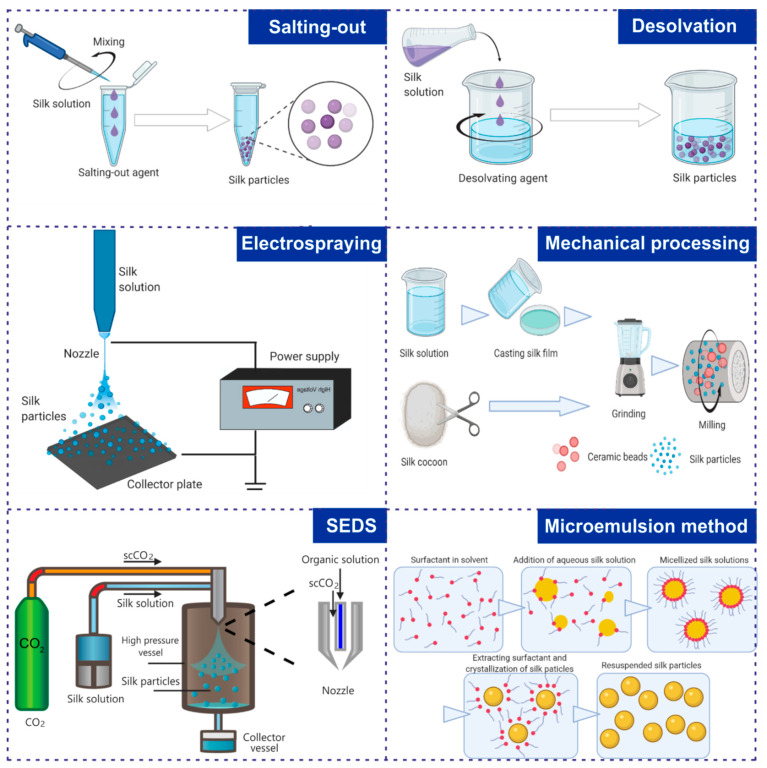
Schematic summary of techniques for the preparation of silk-based nanoparticles. Created with BioRender.com accessed on 28 June 2021.

A few review papers that describe in detail the fabrication methods of silk nanoparticulate carrier systems were published recently [223,232]. Figure 3 shows a schematic representation of the most commonly used silk sphere formation methods.

## 8. Conclusions and Future Perspectives

The ability to customize material properties makes silk a promising biomaterial for drug delivery platforms in cancer treatment and beyond. Favorable features characterize silks compared to other tested materials, such as biodegradation, nontoxicity, and low immunogenicity. Moreover, silks offer a variety of structural morphologies of formed biomaterials. Although fibrous structures can be found in nature, humans can produce silk materials of various shapes adapted to the demand. Due to this variability, silk may be considered when designing carriers that can deliver drugs systemically or locally.

Although silk-based biomaterials are increasingly capturing the scientific community’s interest, several challenges to be overcome remain. Clinical uses of silk are emerging (e.g., stitching, surgical meshes, and fabrics). Moreover, the selected formats, such as films, scaffolds, electrospun materials, hydrogels, and particles, are tested in clinical trials (e.g., wound healing, tissue engineering) (reviewed in [233,234]). The preliminary studies showed no induction of inflammation, giving great hope for future success. However, current clinical use and in vivo studies are limited mainly to silkworm silk-based materials. Although research related to products made of spider silks are less advanced, a few preclinical reports have indicated that bioengineered spider silk is also safe for biomedical applications [90,101,207,235,236]. However, to our knowledge, no clinical trials using recombinant spider silk products are available.

The recombinant production of silk gives advantages that may significantly increase its potential use. These recombinant silks result from the expression of artificially engineered genes, and genetic engineering offers the possibility of generating various forms of silk genes. Construct variability may concern not only the sequence of the silk itself, which would modify the properties of silk [79,117], but also the silk functionalization. The functionalized bioengineered silk carries a functional group(s) on each silk molecule. Thus, biotechnological production of silk offers one of the most efficient methods for functionalization. This may result in obtaining vehicles with certain specific properties. The affinity to anticancer-related drugs can be accommodated. Silks functionalized for the controlled delivery of chemotherapeutics [125], oligotherapeutics (DNA/RNA-based) [80,82,83,237], and inorganic compounds [126] were investigated. Moreover, functionalized silk carriers may be designed to selectively deliver and release the drug only to cancer cells [84,85,89,90].

However, the scalability of recombinant silk production and carrier preparation techniques may emerge as a challenge in translating in vitro results and preclinical in vivo research to further clinical trials. To apply DDSs based on silk in clinical trials, bioengineered silk proteins must be produced not at a laboratory scale but must be translated into pilot and manufacturing scales. Moreover, the endotoxin-free preparation of recombinant proteins is still very challenging. The complete removal of these byproducts is necessary for the effective and safe implementation of silk materials in patients.

**Figure 4:**
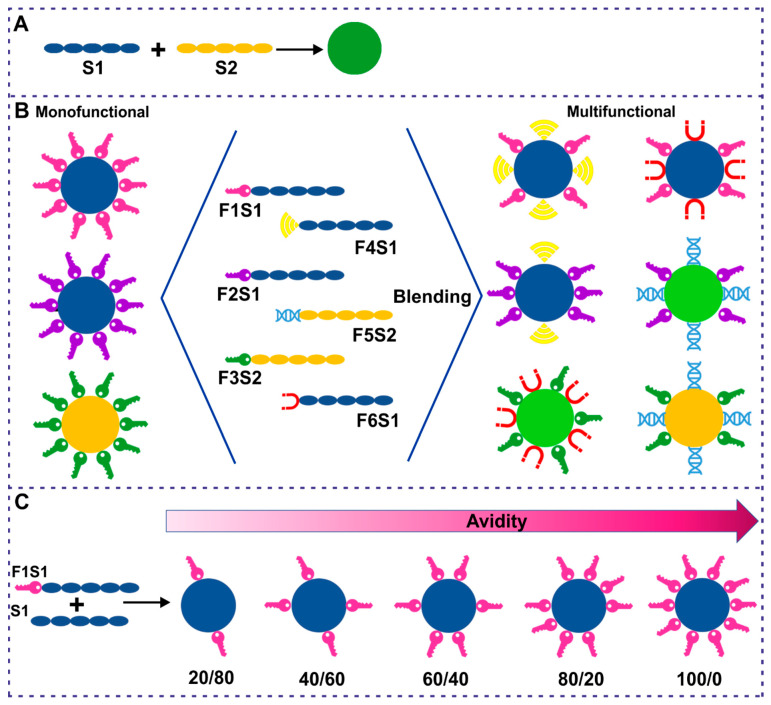
Blending strategy for development of a versatile silk-based drug delivery system. Silk proteins are blended depending on demand before the material (e.g., spheres) formation. (**A**). Blending two types of silk (S1 and S2) to obtain spheres of distinct properties. (**B**). Blending silk proteins of two (or more) distinct functionalization to obtain multifunctional material. (**C**). Blending silk proteins at various ratios to control material’s avidity to cells, drugs, and others molecules.

Although the biotechnological production and purification of silk can be challenging, it offers the possibility of producing various types of silk. We indicated previously that the effectiveness of silk-based drug delivery systems could be increased by blending various silk molecules. The “blending” strategy assumes mixing the silk variants before biomaterial formation. This concept was already studied by using two distinct bioengineered spider silk proteins to obtain a material that combined the properties of both silks (Figure 4A) [71,88]. Moreover, the blending of silks that carried two distinct functionalizations resulted in the formation of spheres that maintained both specific functions (specific cancer cell recognition and drug affinity) [125]. The concept of blending two (or more) types of silk and/or diversely functionalized silks can be further expanded (Figure 4B). The selected variants of silk can be chosen as needed and used to produce carriers that target a specific type of cell(s) or carry a distinct type of drug(s). The function related to the drug affinity can be matched with the function of targeting specific cell(s) in one type of the blended silk carrier (multifunctional particles). Moreover, by changing the mixing ratio of functionalized vs nonfunctionalized silks (or other functionalized silk), the avidity of silk particles can be controlled (Figure 4C). The binding strength of the silk carriers to drugs or cellular receptors can be adjusted as required to enhance the extravasation and tumor penetration properties of silk particles or to regulate the binding and release of therapeutic agent(s).

The possibility of miscellaneous modifications of silk and benefits resulting from blending them allows the design of a DDS that may meet personalized and targeted anticancer therapy requirements. However, one should be aware that some limitations of this strategy may emerge. Due to toxicity problems, not all functional groups may be fused to silk, or a high yield of recombinant production may not be achieved. Moreover, a high number of blended functional groups may increase the toxicity and/or immunogenicity of the silk carriers or their off-target effects. It should be emphasized that in principle, immunogenicity should be tested separately for each silk-based biomaterial. Unwanted immune system interactions may lead to off-target drug release during particle circulation, antibody formation, or induction of inflammation. As a heterogenic group of materials, silk differs in composition and structural characteristics that may affect its immunogenic properties [238]. Various sources of silk, processing methods, and material morphologies should be considered in terms of the body’s response to adjust the properties of the biomaterial to the desired or undesired immune response. As a tunable material, silk may also have the potential for immune-related biomedical applications [238].

**Figure 5:**
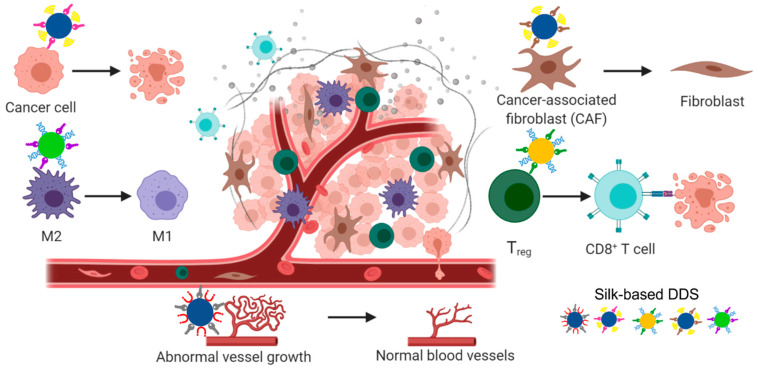
Tumor microenvironment (TME) and potential targets for anticancer therapy. Cancer cells, cancer-associated fibroblasts (CAFs), angiogenesis-related cells (endothelial cells, pericytes), and immunological cells, including tumor-associated macrophages (TAMs/M2) and lymphocytes T_reg_ as potential targets for treatment using silk-based drug delivery system. Created with BioRender.com accessed on 29 June 2021.

The blending strategy for the generation of silk-based DDS can be particularly useful in anticancer therapy. Tumor is not a homogenous tissue and consists of various types of cells (Figure 5). Therefore, it may be advantageous to modify drug carriers to target not only cancer cells but also cells of the tumor microenvironment. Stromal cells, such as cancer-associated fibroblasts (CAFs), angiogenesis-related cells (endothelial cells, pericytes), and immunological cells, including tumor-associated macrophages (TAMs), are potential targets for anticancer therapy. Spheres made of bioengineered MS2KN silk were successfully used as carriers to deliver anticancer oligotherapeutics to macrophages in vitro [80]. Their application as DDSs is currently under investigation in a mouse breast cancer model (data not presented). Anticancer therapy can be envisioned with the simultaneous or successive administration of silk spheres that specifically deliver various drugs targeting different cell types (Figure 5). Moreover, this technology is universal and potentially adaptable to various types of malignances; for example, silk particles developed for immunotherapy that were examined in breast cancer potentially can be used to modify immunosuppressed TME in other cancer types. Additionally, future studies may identify new molecules associated with cancer and TME cells (new potential targets). Such discoveries will probably be accelerated by the development of new bioinformatics tools and computational methods.

The current review focuses on the use of silk delivery systems in oncology. However, the unique silk properties combined with the possibility of their further modifications and blending open the gate to numerous potential biomedical applications, not only in the oncology field. The examples analyzed in context show that silk is a promising material, and we can expect the further development of silk-based biomaterials.

## Figures and Tables

**Table 1:** Various silk fibroin formulations for local delivery of therapeutic agents in cancer treatment.

Biomaterial Format	Released Therapeutic	Target	In Vitro/ In Vivo Model	Findings	Ref
Film	Doxorubicin	Breast cancer	MDA-MB-231/ Orthotopic adrenal tumor xenograft in mice	Sustained release over 4 weeks Doxorubicin release rate could be controlled by manipulating silk crystallinity and beta-sheet content Doxorubicin-loaded silk films significantly greater inhibited primary tumor than intravenously administered drug Silk films loaded with doxorubicin reduced metastatic spread, no local or systemic toxicity	[25]
	Doxorubicin	Neuroblastoma	KELLY, SK-N-AS, IMR-32, SH-SY5Y/ Tumor xenograft in mice	Controlled and sustained drug release up to 30 days Slower tumor growth after treatment with controlled-release silk film Effective treatment by combining surgical resection and local treatment with doxorubicin-loaded films	[148]
	Doxorubicin, Crizotinib	Neuroblastoma	KELLY/ Orthotopic tumor xenograft in mice	Sustained drug release up to 28 days Controllable release kinetics from the silk films by changing the amount and physical crosslinking of silk Intratumoral application of drug-loaded films was more effective in vivo comparing with systemic application of drugs	[167]
	Vincristine, Doxorubicin	ND	ND	Sustained drug release up to 14 days Control over drug release by altering silk film crystallinity and chemical composition	[168]
Fiber mat	Curcumin, Doxorubicin	ND	ND	Dual drug delivery (curcumin-loaded nanoparticles and doxorubicin-loaded core/shell nanofibers) and sustained release Control over the amount of drug release from nanofibers by adjusting the crystal content of nanofibers with the water-annealing process at a different temperature, release up to 40 h	[169]
	Curcumin	Colorectal carcinoma	HCT-116/ Tumor xenograft in mice	Curcumin-loaded nanofibrous matrices had enhanced anti-cancer effect as compared to free drug No toxic effect on normal NCM-460 cells Implantation of curcumin-loaded nanofibrous matrices resulted in tumor growth inhibition in vivo	[158]
Gel	Vincristine, Doxorubicin	Neuroblastoma	KELLY/ Orthotopic tumor xenograft in mice	Dual drug delivery and sustained release of drugs up to 25 days Intratumoral delivery of vincristine and doxorubicin significantly slowed tumor growth and increased drug availability as compared to intravenous administration	[23]
	Vincristine, Doxorubicin	Ewing’s sarcoma	A-673/ Tumor xenograft in mice	Combination of vincristine-loaded silk gels and doxorubicin-loaded silk foams Delivery of vincristine inside the sarcoma tumor with silk gel decreased tumor growth more effectively compared to silk foam	[170]
	Vincristine	Neuroblastoma	KELLY/ Orthotopic tumor xenograft in mice	Sustained release silk gels, Multiple injections of vincristine-loaded silk gels suppressed tumor growth Tumor growth more significantly suppressed by distributed injections compared to central injections of drug-loaded silk gel	[140]
Hydrogel	Doxorubicin	Breast cancer	MDA-MB-231, MCF-7/ Tumor xenograft in mice	Controlled doxorubicin release Doxorubicin-loaded silk hydrogels reduced primary and metastatic tumors growth Reduced toxicity compared to systemic drug administration	[26]
	Doxorubicin	Breast cancer	MDA-MB-231/ Tumor xenograft in mice	Silk hydrogels displayed thixotropic capacity allowing for easy injectability Sustained drug release over 8 weeks Dox-loaded silk hydrogels had a superior antitumor response in vitro and in vivo than free Dox	[171]
Foam	Vincristine	Neuroblastoma	KELLY/ Orthotopic tumor xenograft in mice	Sustained drug release from the foam format over 21 days	[24]
	Vincristine, Doxorubicin	Neuroblastoma	KELLY/ Orthotopic tumor xenograft in mice	Sustained drug release High drugs concentrations within the tumor resulting in slower tumor growth with less post-treatment side effects than equivalent systemic chemotherapy	[23]
Reservoir	Anastrozole	ND	ND/ Sprague-Dawley rats	Biocompatibility of silk reservoir rods Sustained drug delivery for 91 days measured in a pharmacokinetic study in vivo Biodegradation profile suitable for long-term sustained delivery of breast cancer therapeutics	[139]
	Cisplatin	Neuroblastoma	KELLY/ Orthotopic tumor xenograft in mice	Controlled release of the drug up to 30 days Intratumoral implantation of silk reservoirs decreased tumor growth significantly when compared to free cisplatin	[149]
Wafer	Etoposide	Neuroblastoma	KELLY/ Orthotopic tumor xenograft in mice	Silk coated 6% wafers released the drug up to 45 days, while uncoated wafers for 30 days Intratumoral implantation was effective at decreasing tumor growth. Etoposide-loaded silk wafers induced tumor necrosis	[138]
	Vincristine	Neuroblastoma	KELLY/ Orthotopic tumor xenograft in mice	Sustained drug release from the wafer reservoir for 7 weeks Intratumoral injection slowed tumor growth and increased drug availability as compared to intravenous administration	[24]
Microneedles	Doxorubicin, Rhodamine, ICG	Cervical cancer	HeLa/ Live mouse skin	Microneedles fabricated using a PDMS mold packed with a fibroin scaffold Controlled release up to 144 h More rapid release of doxorubicin from the microneedles with a higher proportion of sucrose Tumor cell viability decreased faster under higher sucrose content in the applied microneedles The soluble sucrose content and fibroin scaffold within microneedles accelerated the transdermal release of the photothermal agent in vivo	[137]

Dox, doxorubicin; ICG, indocyanine green; PDMS, polymethylsiloxane; ND, not determined.

**Table 2:** Delivery of chemotherapeutics using silk-based particles.

Drug	Silk Source	Preparation Method	Particle Size	Characterization	Functionalization/Surface Modification	Target/ In Vitro/ In Vivo Model	Outcome/Findings	Ref
Doxorubicin	*B. mori* silk fibroin	Desolvation with acetone	100 nm	SEM, DLS, Zeta potential Drug loading/release Cellular uptake (CLSM) Cytotoxicity		Breast cancer/ MCF-7 MCF-7-ADR/ND	pH-dependent drug release up to 6 days Enhanced endocytic uptake and lysosomal accumulation	[174]
		Nanoprecipitation with acetone	106 nm	Size, Zeta potential SEM Encapsulation efficiency Cytotoxicity		Breast cancer/ MDA-MB-231/ ND	Simple, quick and reproducible method of particle preparation High drug encapsulation efficiency Sustained drug release	[175]
		Electrospraying with PVA blends	600–1800 nm	DLS, Zeta potential SEM, TEM Drug loading/release Cytotoxicity (MTT) Apoptosis assay In vivo study		Breast cancer/ MDA-MB-231/ tumor xenograft in mice	Very good monodispersity High drug encapsulation efficiency Controlled drug release for 72h External ultrasound triggered and accelerated drug release	[176]
		Silk/PVA phase separation within microfluidics device	2.8–6.8 µm	SEM Drug loading/release Cytotoxicity (MTT) Macrophage activation Cellular uptake (CLSM)		Neuroblastoma/ KELLY THP-1/ ND	High drug loading capacity and efficiency pH-dependent drug release Sustained drug release over 23 days Uptake by THP-1 monocytes Macrophage activation in response to silk particle exposure	[177]
		Salting-out with potassium phosphate	530 nm	Size, SEM, Zeta potential, FTIR, BET analysis (porous structure) Cytotoxicity (CCK-8) Cellular binding and internalization (FCM, CLSM)	FA-conjugated	Cervical cancer/ HeLa/ ND	FA-targeted and pH-responsive particles Controlled drug release up to 32 h Enhanced internalization in cancer cells overexpressing FA receptor Higher cytotoxicity against HeLa cells than particles without functionalization	[178]
		Acetone nanoprecipitation	116 nm	DLS, Zeta potential, SEM, FTIR, Drug loading/release Macrophage activation Cytotoxicity (MTT) Cellular uptake (CLSM)	PEGylated silk	Breast cancer/ MCF-7/ ND	Increased particle stability Increased clearance time than non-modified particles High drug entrapment efficiency and release capacity pH-dependent drug release over 14 days	[179]
	*A. pernyi* silk fibroin	Ion-induced self- assembly	100–500 nm	Size, Zeta potential, SEM, FTIR, XRD, Drug release Cytotoxicity (Alamar blue)		Liver cancer/ HepG-2/ ND	Self-assembly induced by cations (Na+, Ca2+, and Ce3+) RGD-containing silk fibroin material pH-sensitive and sustained drug release up to 11 days	[180]
		Self-assembly	30–1000 nm	SEM, FTIR, XRD Drug loading/release		ND	pH-sensitive and sustained release for over 23 days	[181]
	*A. mylitta* silk fibroin	Desolvation with acetone	150–170 nm	TEM, DLS, Zeta potential Drug loading/release Cellular binding and internalization (FCM, CLSM) Cytotoxicity (MTT) Macrophage activation	FA-conjugated	Breast cancer/ MDA-MB-231/ ND	Capable of sustained drug release up to 21 days Selective cancer cells targeting Enhanced cellular binding and uptake via endocytosis than non-functionalized particles	[182]
	*B. mori* silk sericin-chitosan	Two-step crosslinking with chitosan and EDC	200–300 nm	Drug loading/release Zeta potential Cytotoxicity (CCK-8) Hemolysis assay Plasma coagulation assay In vivo studies		Breast cancer/ MCF-7 and Liver cancer/ HepG-2/ tumor xenograft in mice	Excellent colloidal stability Stable in the absence of cryoprotectants Biocompatible in animal study Low systemic toxicity of the released drug	[44]
	*A. pernyi* silk sericin	Silk-templated hydroxyapatite (HAp) mineralization	1.2 µm	SEM, TEM, DLS, FTIR, XRD Drug loading/release Cryo-SEM Cytotoxicity (Alamar blue) Cellular uptake (CLSM)		Breast cancer/ Bcap-37 and Cervical cancer/ HeLa/ ND	Uniform and porous microparticles pH-responsive characteristic due to the presence of pH-responsive HAp Controlled and sustained release of drug	[46]
	Bioengineered silk (SELP)	Self-assembly with hydrophobic Dox	50–142 nm	DLS, Drug loading/release Cytotoxicity (MTT) Cellular binding (FCM) and uptake (CLSM)		Cervical cancer/ HeLa/ ND	Fabricated and loaded with an aqueous process under mild conditions Simple method to control particle size High uptake of the nanoparticles by the cancer cells Internalization of the nanoparticles through endocytosis	[37]
	Bioengineered *N. clavipes* spider silk (MS1)	Salting-out with potassium phosphate	300–400 nm	Size, Zeta potential, SEM, FTIR, Drug loading/release Cellular binding (FCM) and uptake (CLSM) Cytotoxicity (MTT)	H2.1 and H2.2 peptides-conjugated (anti-Her2)	Breast cancer/ SKBR-3 and Ovarian cancer/ SKOV-3/ ND	pH-dependent drug release up to 15 days Enhanced targeted binding to Her2-overexpressing cells Enhanced internalization into targeted cancer cells Higher toxicity towards cancer cells than control cells No cytotoxic	[88,89]
				In vivo studies (toxicity, biodistribution, efficiency)	H2.1 peptide-conjugated	Breast cancer/ murine D2F2 and D2F2E2/ tumor in mice	Enhanced tumor-specific targeting in vivo than non-functionalized particles No systemic toxicity as compared to free Dox Suppression of cancer cell growth in vivo	[90]
	Bioengineered *N. clavipes* spider silk (MS1, MS2)	Salting-out with potassium phosphate	<400 nm	Size, SEM, Drug loading/release Cellular binding (FCM) and uptake (CLSM) Cytotoxicity (MTT)	H2.1 peptide/ DOX binding peptide-conjugated	Breast cancer/ SKBR-3/ ND	pH-dependent drug release up to 7 days Double functionalization of silk spheres for controlled Dox delivery into Her2-positive cancer cells Enhanced targeted binding and internalization into Her2-overexpressing cells Higher drug-loading capacity, binding per cell and cytotoxic effect compared with control spheres, Higher toxicity towards cancer cells than control cells	[125]
Paclitaxel	*B. mori* silk fibroin	Desolvation with ethanol and freezing	270–520 nm	Size, Zeta potential, FTIR, HRSEM, TEM Drug loading/release		ND	Easy and mild method of particle preparation Particles with controllable shape and size Drug release for over 9 days	[183]
		Desolvation with ethanol	158–206 nm	Size, Zeta potential, TEM, FTIR, XRD, Drug loading/release Cellular binding (microscopy) Cytotoxicity (MTT) Apoptosis assay In vivo studies (toxicity, efficiency)		Gastric cancer/ BGC-823 and SGC-7901/ Tumor xenograft in mice	Sustained drug release for 100 h Drug-induced cytotoxicity when incorporated into nanoparticles Excellent antitumor efficacy in mice No systemic toxicity	[184]
		Desolvation with ethanol	100–600 nm	Size, Zeta potential TEM Drug loading/release Cellular uptake (CLSM) Cytotoxicity		Cervical cancer/ HeLa and Liver cancer/ HepG-2/ ND	Dual drug loading (Ptx, Dox) Controlled and sustained drug release for over 7 days High cellular uptake via endocytosis Suppression of cancer cell growth in vitro	[185]
		Desolvation with acetone	115 nm	DLS, SEM, FTIR Drug loading (UHPLC-MS/MS) Cytotoxicity (MTT)		Pancreatic cancer/ CFPAC-1/ ND	The drug-encapsulation in nanoparticles did not influence its cytotoxicity profile High dose-dependent cytotoxic activity of drug-loaded nanoparticles	[186]
		Desolvation with ethanol	186 nm	Size, Zeta potential, FTIR, TEM, Cytotoxicity (MTT) Cellular binding (fluorescence microscopy) In vivo study (biodistribution, efficiency)	Anti-iRGD-EGFR-conjugated	Cervical cancer/ HeLa/ Tumor xenograft in mice	High drug content and loading efficiency Enhanced tumor-specific targeting in vitro and in vivo than non-functionalized particles Good antitumor effect	[187]
	*A. mylitta* silk sericin	Self-assembly with pluronic surfactants	100–110 nm	DLS, TEM Fluorescence microscopy Cytotoxicity (MTT) Apoptosis assay (FCM, CLSM, western blot)		Breast cancer/ MCF-7/ ND	High loading of hydrophobic drug Stable in aqueous solution High cellular uptake Efficient cytotoxicity towards cancer cells when loaded with drug	[43]
Cisplatin	*B. mori* silk fibroin	Electrospraying	59 nm	SEM, DLS, FTIR Drug loading/release Cytotoxicity (MTT) Apoptosis assay (FCM)		Lung cancer/ A-549/ ND	Drug release for more than 15 days Internalization into cancer cells Sustained and efficient killing of cancer cells Low toxicity in fibroblasts	[188]
		Spray-drying/spray-freeze-drying and crosslinking with genipin	10.8–22.75 µm	DLS, SEM, AFM, XRD Aerosolization (NGI) Drug release Cytotoxicity (CCK-8, PicoGreen) Cell migration and invasion		Lung cancer/ A-549/ ND	Drug loading with or without cross-linking showing different release profiles Drug delivery directly to the lungs via powder inhalers Enhanced cytotoxicity when drug was delivered using the cross-linked particles	[189]
		Precipitation with ionic liquids and high-power ultrasounds	173 nm	DLS, TEM, XRD Drug loading/release Cytotoxicity (MTT) Apoptosis assay (flow cytometry)		Ovarian cancer/ A-780 and A-780-cisR and Breast cancer/ SK-BR-3, MCF-7 and MDA-MB-231/ ND	Efficient loading with Pt(IV) prodrug PtBz High cellular uptake Overcame drug resistance to cisplatin	[190]
5-Fluorouracil	*B. mori* silk fibroin	Desolvation with acetone	278.2–364.9 nm	Drug loading/release Cytotoxicity (CCK-8) Degradation Cellular uptake (CLSM) In vivo studies (toxicity, biodistribution, efficiency)	cRGDfk and Ce6-conjugated	Gastric cancer/ MGC-803/ tumor xenograft in mice	Targeted drug delivery and PDT Active tumor targeting Together with laser irradiation, the drug-loaded particles reduced the tumor burden Biocompatibility and safety in vivo	[127]
		Desolvation and crosslinking with genipin	217 nm	TEM, DLS, FTIR Drug loading/release Apoptosis assay (FCM) In vivo studies (toxicity, efficacy)		Murine breast cancer/ 4T1/ tumor-bearing mice	Binary drug loading (5-FU and curcumin), High loading efficacy Improvement in the cytotoxic activity and bioavailability compared with free drugs Toxic effect toward cancer cells in vitro and in vivo The anticancer effect observed may be induced by the apoptosis of cells via the generation of cellular ROS	[191]
		Desolvation with acetone	220 nm	DLS, Zeta potential, SEM, TEM, FTIR, XRD, Drug loading/release Cytotoxicity (MTT)		Breast cancer/ MCF-7 Colon cancer/ HT-29	High loading efficiency Controlled and sustained drug release Enhanced cytotoxic effect on cancer cells	[192]
	*B. mori* pupa protein (Pp)	Desolvation with ethanol	162 nm	FTIR, Size, Zeta potential Drug loading/release Cytotoxicity (hemolysis assay, MTT) In vivo studies (toxicity, biodistribution, efficacy,)		Lymphoma/ DAL/ tumor-bearing mice	Particles that are easy to prepare, modify, with good biocompatibility and bio-adhesivity High entrapment efficiency and capacity Sustained drug release Anticancer efficiency in vivo without causing toxicity in the healthy tissue	[193]
FUDR	*B. mori* silk fibroin	Desolvation with ethanol and freezing	210–510 nm	Size, Zeta potential, SEM, TEM, Drug loading/release Cytotoxicity (MTT) Cellular uptake (CLSM)		Cervical cancer/ HeLa/ ND	Controllable shape and size, without apparent aggregation Drug release time over 2 days Cancer cells growth inhibition Similar curative effect to kill or inhibit Hela cells to the free drug	[194]
Methotrexate	*B. mori* silk fibroin	Suspension-enhanced dispersion by supercritical CO_2_ (SEDS)	112 nm	FTIR, SEM Drug loading/release Cellular uptake (CLSM)		Skin from guinea pig	High drug loading efficiency Magnetic nanoparticles for transdermal drug delivery Improved penetration of drugs across the skin	[195]
	*B. mori* silk fibroin-albumin	Desolvation with acetone and crosslinking with glutaraldehyde	152–176 nm	TEM, DLS, Zeta potential, FTIR, Drug loading/release Cellular uptake (CLSM) Cytotoxicity (MTT, hemolysis assay)		Breast cancer/ MDA-MB-231/ ND	Silk-albumin conjugates High drug loading efficiency Sustained drug release over 12 days	[109]
Gemcitabine	*B. mori* silk fibroin	Desolvation with DMSO	302 nm	DLS, SEM, Zeta potential Cytotoxicity (MTT) Cellular uptake (CLSM) In vivo studies (biodistribution, toxicity, efficiency)	SP5-52 peptide-conjugated	Lung cancer/ LL/2/ tumor-bearing mice	Targeted delivery to lung cancer cells Higher cellular uptake and cytotoxicity in cancer cells in vitro than non-modified particles Increased accumulation in lung tissue than non-modified particles The improved therapeutic outcome in vivo and minimized systemic toxicity than free drug	[124]

PVA, poly(vinyl alcohol); DLS, dynamic light scattering; SEM, scanning electron microscopy; TEM, transmission electron microscopy; AFM, atomic force microscopy; FTIR, Fourier-transform infrared spectroscopy; XRD, X-ray diffraction; CLSM, confocal laser scanning microscopy; FCM, flow cytometry; EDC, ethylcarbodiimide; FA, folic acid; PEG, polyethylene glycol; HAp, hydroxyapatite; SELP, silk-elastin-like polymer; Dox, doxorubicin; Ptx, paclitaxel; 5-FU, 5′-fluorouracil; FUDR, floxuridine; Ce6, chlorin e6; PDT, photodynamic therapy; ROS, reactive oxygen species; DMSO, dimethyl sulfoxide.

**Table 3:** Delivery of anticancer therapeutic agents using silk-based nanoparticles.

Type of Anticancer Therapeutic	Therapeutic Agent	Silk Source	Preparation Method	Particle Size	Functionalization/Surface Msodification	Target/ In Vitro/ In Vivo Model	Outcome/Findings	Ref
Plant-derived therapeutic agents	Curcumin	*B. mori* silk fibroin	Precipitation with ionic liquids and high-power ultrasounds	166–171 nm		Liver cancer/ Hep3B and Neuroblastoma/ KELLY/ ND	Sustained drug release up to 3 days Drug bioavailability Cytotoxic activity towards cancer cells No toxic effect in healthy cells	[196]
			Suspension-enhanced dispersion by supercritical CO_2_ (SEDS)	<100 nm		Colon cancer/ HCT-116/ ND	Time-dependent intracellular uptake ability Improved inhibition effects on colon cancer cells No toxic effect in healthy cells	[197]
			Desolvation and cross-linking with genipin	217 nm		Murine breast cancer/ 4T1/ Tumor in mice	Binary drug loading (5-FU and curcumin) High loading efficacy Improvement in the cytotoxic activity and bioavailability compared with free drugs Toxic effect toward cancer cells in vitro and in vivo	[191]
		*B. mori* silk fibroin-chitosan blend	Microdot capillary method	<100 nm		Breast cancer/ MCF-7 and MDA-MB-453/ ND	Sustained drug release over 9 days Efficacy against Her2-overexpressing cancer cells	[198]
	Resveratrol	*B. mori* silk sericin	Desolvation with DMSO and pluronic F-68	200–400 nm		Colon cancer/ Caco-2/ ND	High drug encapsulation levels and stable drug release profile over 72 h High intra-cellular internalization efficiency The anticancer effect, but no toxicity towards healthy cells	[199]
	Triptolide/ celastrol	*B. mori* silk fibroin	Desolvation with acetone and ethanol	166 nm/ 170 nm		Pancreatic cancer/ MIA PaCA-2 and PANC-1/ ND	Improved bioavailability and pharmacokinetic properties compared to free drugs The pH-dependent sustained drug release over 192 h Increased therapeutic efficiency compared to free drugs	[200]
	Emodin	*B. mori* silk fibroin	Lyophilisation of silk fibroin with emodin-loaded liposomes	316 nm		Breast cancer/ MCF-7, BT-474 and MDA-MB-453/ ND	Silk coating of liposomes decreased drug release rate compared to uncoated liposomes Longer intracellular retention of silk coated liposomes than liposomes w/o coating lead to the longer availability of emodin for down-modulation of various Her2/neu pathways	[201,202]
	α-mangostin	*B. mori* silk fibroin	Desolvation and crosslinking with EDC or PEI	300 nm		Colon cancer/ Caco-2 and Breast cancer/ MCF-7/ ND	Increase in water solubility of the drug Maintained α-mangostin’s apoptotic effect Increased cytotoxic effect on cancer cells than the free drug Reduction of hematoxicity compared to free drug	[203]
Nucleic acid-based therapeutic agents	siRNA (anti-LUC)	*B. mori* silk fibroin-oligochitosan blend	Desolvation with acetone	250–450 nm		Lung cancer/ H1299/ ND	Enhanced particle loading capacity than oligochitosan polyplexes Enhanced serum stability of siRNA than naked nucleic acid Increased gene silencing effect compared with oligochitosan polyplexes	[27]
	pDNA encoding GFP	*A. pernyi* silk fibroin (ASF)	Self-assembly with PEI/DNA complexes	230–360 nm		Colon cancer/ HCT-116/ ND	PEI/DNA complexes coated with RGD-rich ASF Increased target specificity in comparison with PEI/DNA complexes alone Higher uptake of silk coated complexes in cancer cells due to the affinity of the RGD peptides from ASF for integrins, Lower post-transfection cell toxicity than uncoated complexes	[204]
	siRNA (anti-CK2, anti-ASH2L, anti-Cyclin D1)	*B. mori* silk sericin-albumin	Desolvation with ethanol	127–142 nm	poly-L-lysine (PLL)-conjugated and hyaluronic acid (HA)-conjugated	Laryngeal cancer/ Hep-2/ ND	Particles modified with PLL for siRNA binding and decorated HA to target cancer cells High siRNA entrapment Downregulation of target CK2, ASH2L and Cyclin D1 genes Higher silencing effect comparing with naked siRNA	[205]
	siRNA (anti-STAT3)	Bioengineered *N. clavipes* spider silk (MS2KN)	Salting out with potassium phosphate	202 nm	poly-L-lysine (KN)	Macrophages/ J774/ ND	Approach for cancer immunotherapy Protection of CpG-siRNA therapeutics from degradation by serum nucleases CpG-STAT3-siRNA targeted delivery to TLR9-positive macrophages Prolonged siRNA presence in macrophages than naked siRNA Prolonged silencing effect on STAT3 expression than naked siRNA	[80]
	pDNA encoding LUC	Bioengineered *N. clavipes* spider silk (15mer)	Self-assembly with pDNA	186 nm	poly-L-lysine and RGD-conjugated	Cervical cancer/ HeLa/ ND	High pDNA delivery efficiency Increased integrin-mediated transfection with RGD sequences than non-conjugated constructs	[82]
				99 nm	poly-L-lysine and ppTG1-conjugated	Melanoma/ MDA-MB-435/ ND	High transfection rates Controlled enzymatic degradation rate of the silk-based pDNA complexes enables the regulation of the release profile of genes from the complexes	[83]
				90 nm	poly-L-lysine and Lyp1 or F3 peptide-conjugated	Melanoma/ MDA-MB-435 and Breast cancer/ MDA-MB-231/ Tumors in mice	Enhanced pDNA delivery than non-functionalized complexes Low cytotoxicity Functionalization with F3 tumor homing peptide was the most effective in pDNA delivery to cancer cells	[84]
Protein-based therapeutic agents	Lactoferrin	*S. cynthia* ricini Eri silk	Milling	200–300 nm		Breast cancer/ MCF-7 and MDA-MB-231/ ND	Sustained release of therapeutic agents Higher stability in presence of proteolytic enzymes than bovine lactoferrin alone EGFR or TfR2 receptors-mediated endocytosis of nanoparticles Cytotoxic properties towards cancer cells	[206]
	Peptides from ovoalbumin (C16-OVA)	Recombinant *A. diadematus* spider silk	Salting-out with potassium phosphate using micromixing device	369–386 nm		Bone marrow derived cells (BMDC)/ in vivo mouse model	Potential approach for cancer vaccine immunotherapy Preferential uptake by immunological cells Localization in lysosomes Particles cleaved by lysosomal cathepsins to release transported peptide Antigen-specific proliferation of T-cells and cytotoxicity of released peptides in vivo	[207]
Inorganic agents	IONPs/ Dox	Bioengineered *N. clavipes* spider silk (MS1, MS2, EMS2)	Salting-out with potassium phosphate	500 nm		ND	The addition of silk did not influence magnetic properties of IONPs Efficient incorporation and sustained release of incorporated drug (Dox) Not cytotoxic in vitro	[110]
				ND	H2.1 peptide-conjugated (anti-Her2)	Breast cancer/ SK-BR-3/ ND	Specific affinity of functionalized magnetic silk particles towards Her2-overexpressing cancer cells, Efficient binding of doxorubicin Ability to generate heat upon application of magnetic field (MF) Induction of hyperthermia in targeted cancer cells	[126]
	IONPs/ Dox	*B. mori* silk fibroin	Salting-out with potassium phosphate	171–206 nm		Breast cancer/ MCF-7 and MCF-7-ADR/ tumor xenograft in mice	High drug loading efficiency pH-dependent drug release up to 4 days Efficient magnetic targeting and intracellular delivery into both MCF-7 and MCF-7/ADR Ability to overcome multidrug resistance (MDR) The magnetic targeting to tumor in vivo	[208]
	IONPs/ Mtx		Suspension-enhanced dispersion by supercritical CO_2_ (SEDS)	112 nm		Skin from guinea pig (ex vivo studies)	High drug loading efficiency Magnetic nanoparticles for transdermal drug delivery Improved penetration of drugs across the skin upon application of MF	[195]
	IONPs/ Cur		Salting-out with potassium phosphate	90–350 nm		Breast cancer/ MDA-MB-231/ ND	The magnetic targeting to cancer cells in vitro Higher uptake of drug-loaded nanoparticles than free drug Lower viability of cancer cells than control cells	[209]
	IONPs/ ODN (anti-c-myc)	*B. mori* silk fibroin mixed with PEI	Salting-out with sodium phosphate	<200 nm		Breast cancer/ MDA-MB-231/ ND	Magnetic-silk/PEI core-shell nanoparticles Lower surface charge and reduced cytotoxicity than magnetic-PEI-coated particles High cellular uptake, efficient magnetofection level	[210]
	MnO_2_/ Dox/ICG	*B. mori* silk fibroin	Self-assembly induced by organic solvent	140 nm		Breast cancer/ 4T1/ tumor-bearing mice	Strong and stable photothermal effect upon NIR irradiation Effective tumor-specific accumulation via EPR effect Combination chemotherapy, PDT and PTT under the guidance of NIR/MR imaging Reduced systemic toxicity	[211]
Photo-sensitive or photo-dynamic agents	ICG	*B. mori* silk fibroin	Desolvation with acetone	210 nm		Glioblastoma/ C6/ Tumor xenograft in mice	A therapeutic nano-platform for imaging and PTT of glioblastoma High encapsulation efficiency of photosensitive agent and slow drug release profile in vitro Increased stability of PTT effect under NIR irradiation than free drug Internalization of particles by cancer cells in vitro Accumulation of particles in site of tumor and tumor growth suppression in vivo	[212]
	Ce6/ 5-FU	*B. mori* silk fibroin	Desolvation with acetone	278.2–364.9 nm	cRGDfk and Ce6-conjugated	Gastric cancer/ MGC-803/ Tumor xenograft in mice	Combination of targeted drug delivery and (PDT) Active tumor targeting of integrin receptor-overexpressing cells Together with laser irradiation, the drug-loaded particles reduced the tumor burden Biocompatibility and safety in vivo	[127]
	IR780	*B. mori* silk sericin-cholesterol	Self-assembly induced by DMSO	105 nm	FA-conjugated	Gastric cancer/ BGC-823/ ND	Efficient incorporation of photosensitive substance IR780 Improved photo-stability and water solubility of IR780 Efficient absorption by FA-positive cancer cells Excellent PDT and PTT cytotoxicity towards cancer cells under NIR irradiation	[47]

EDC, ethylcarbodiimide; PEI, polyethylenimine; FA, folic acid; Dox, doxorubicin; 5-FU, 5′-fluorouracil; Ce6, chlorin e6; DMSO, dimethyl sulfoxide; CK2, casein kinase II; ASH2L, ASH2 like, histone lysine methyltransferase complex; GFP, green fluorescent protein; LUC, firefly luciferase; PLL, poly-L-lysine; HA, hyaluronic acid; IONPs, iron oxide nanoparticles; ICG, indocyanine green; Mtx, methotrexat; Cur, curcumin; MR, magnetic resonance; NIR, near-infrared, PDT, photodynamic therapy; PTT, photothermal therapy.
